# An Endocrine Jaw Lesion: Dentist Perspective in Diagnosis

**DOI:** 10.1155/2016/2582038

**Published:** 2016-11-15

**Authors:** Lavanya Kalapala, Surapaneni Keerthi sai, Suresh Babburi, Aparna Venigalla, Soujanya Pinisetti, Ajay Benarji Kotti, Kiranmai Ganipineni

**Affiliations:** ^1^Department of Oral & Maxillofacial Pathology, Drs. Sudha & Nageswara Rao Siddhartha Institute of Dental Sciences, Chinoutpalli, Gannavaram, India; ^2^Government Dental College, Vijayawada, India

## Abstract

Brown tumor is a rare nonneoplastic focal giant cell lesion that occurs in hyperparathyroidism patients with a prevalence rate of 0.1% in jaws. We report an extremely rare case of brown tumor in mandible of a 40-year-old female patient that presented as the first clinical manifestation of hyperparathyroidism. Dentist played a pivotal role in the present case by the early diagnosis of lesion and its intervention.

## 1. Introduction

Hyperparathyroidism (HPT) is an endocrine disorder occurring due to increased secretion of paratharmone resulting in a complex of clinical, anatomical, and biochemical alterations [1]. HPT is categorized into 4 types: primary HPT is caused by parathyroid adenomas (85%), hyperplasias (10%), and carcinomas (5%). Secondary HPT occurs as a compensatory increase in paratharmone levels due to hypocalcemia or vitamin D deficiency. Tertiary HPT presents in patients with long-standing secondary HPT resulting in autonomous functioning of parathyroid gland. Fourth type is an ectopic variant seen in patients with other malignancies [2]. Many a times, hyperparathyroidism is discovered accidentally on routine biochemical and radiological investigations [3].

One of the skeletal lesions observed in HPT is brown tumor [4], also termed as Von Recklinghausen's disease of bone or osteitis cystica fibrosa. Due to the presence of excessive hemorrhage, vascularization, and hemosiderin deposits grossly, a characteristic brown color is attained and thus the name “BROWN TUMOR” is derived [5]. However, the term is a misnomer since it is not a true neoplasm [6].

Brown tumor is mostly asymptomatic, but occasionally it may present as a painful exophytic mass [1]. Radiographically it appears as a unilocular or multilocular lesion with an irregular periphery. Histologically it is a focal giant cell lesion which shows multinucleated giant cells within a fibrovascular stroma admixed with areas of hemorrhage and hemosiderin deposits [7].

We report a rare case of brown tumor occurring in mandible of a 40-year-old female patient that was the first clinical manifestation and presented as a multilocular radiolucency, which on further biochemical assessment confirmed the diagnosis of adenoma of parathyroid. Along with this case report other giant cell mimickers of oral cavity are also discussed.

## 2. Case Report

A 40-year-old female reported to the outpatient department with a chief complaint of pain in the lower left back tooth region since 6 months and associated swelling since 3 months. The swelling was initially small in size and gradually attained present size. Patient gave a history of weight loss since 1 year and traumatic incident 3 months before. Patient was hypertensive since 3 months and is under medication.

**Figure 1:**
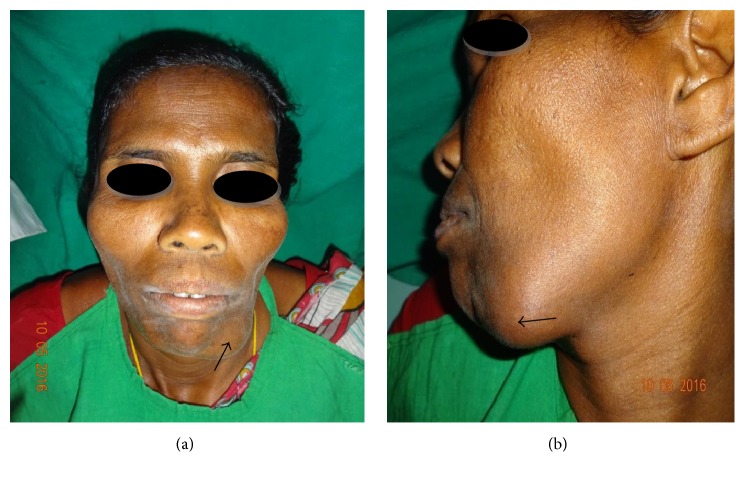
(a, b) Swelling in the left lower side of the mandible.

**Figure 2:**
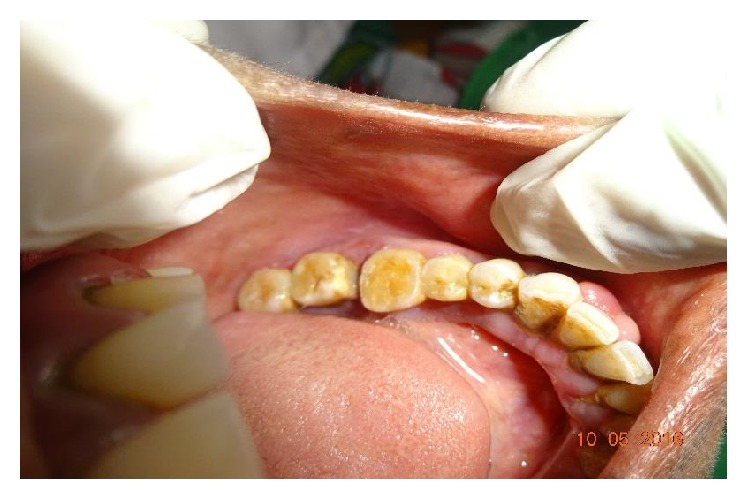
Intraoral swelling with no obliteration of sulcus.

Extraorally, a swelling was observed on the left lower third of the face (Figures 1(a) and 1(b)) and on intraoral examination a swelling of 1 × 3 cm was observed extending from distal aspect of 34 to mesial aspect of 37 with no sulcus obliteration and associated tooth mobility. Overlying mucosa was normal (Figure 2). On palpation the swelling was hard and tender.

**Figure 3:**
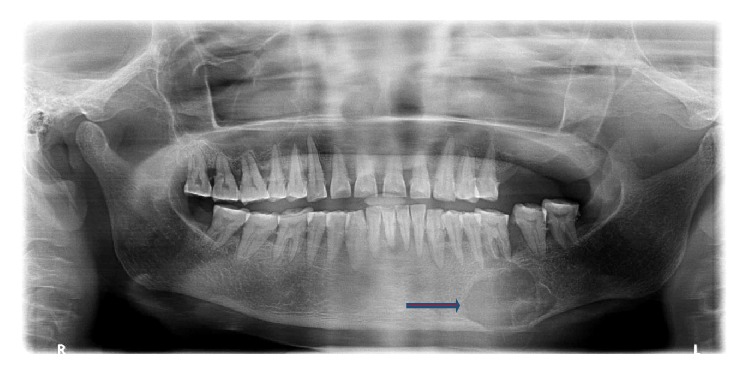
Radiolucent lesion extending from 34 to 37.

OPG revealed a multilocular radiolucent lesion with well-defined margins was seen in relation to 35 and 36 with thinning out of inferior border of mandible. Loss of lamina dura in relation to 35 and 36 along with loss of continuity of mandibular canal was also observed (Figure 3).

**Figure 4:**
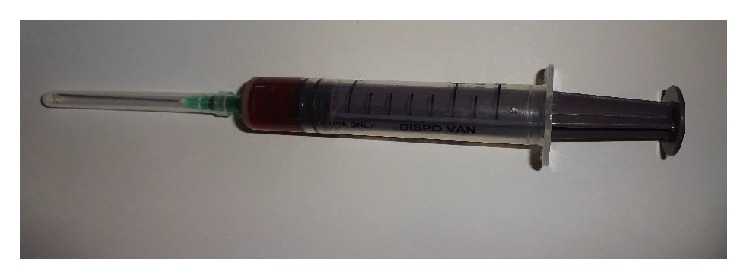
Aspirated fluid.

FNAC revealed a reddish colored aspirate (Figure 4), composed of RBCs, lymphocytes, and neutrophils. Incisional biopsy was done and sent for histopathological evaluation.

**Figure 5:**
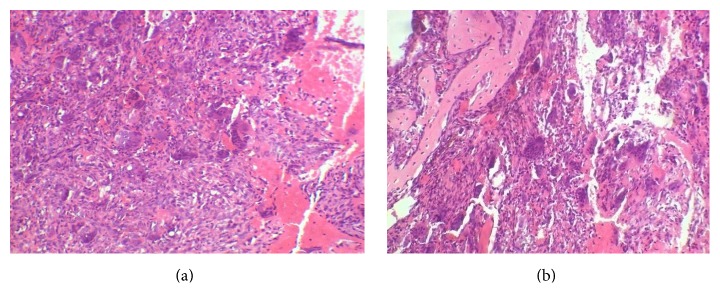
(a) Photomicrograph of 10x view shows numerous multinucleated giant cells and hemorrhagic areas. (b) Photomicrograph of 40x view shows multinucleated giant cells of varying size and shape and areas of osteoid.

On microscopic examination, numerous osteoclast like multinucleated giant cells of varying sizes and shapes which were composed of 10–20 nuclei and dispersed in the background of mononuclear spindle shaped stromal cells were seen. Areas of osteoid, trabecular bone, hemorrhage, and inflammatory component were seen (Figures 5(a) and 5(b)). A giant cell lesion was diagnosed. But to rule out any metabolic disorders, the patient was advised a series of further investigations.

**Figure 6:**
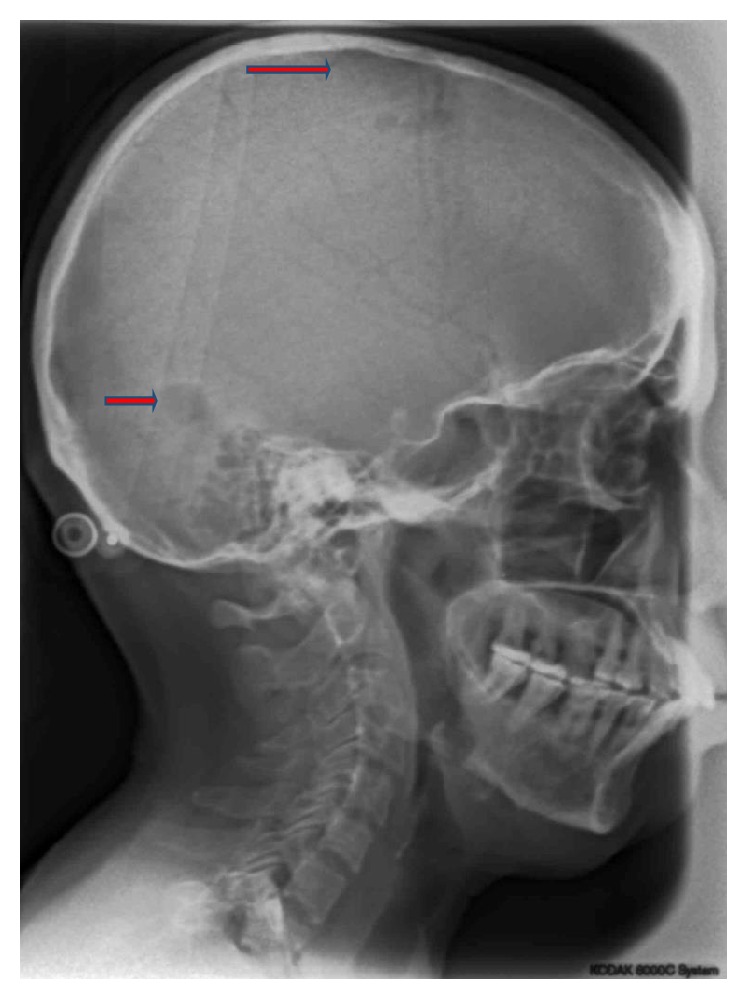
Skull radiograph showing osteolytic areas.

Hematological investigations demonstrated elevated serum calcium and phosphorus levels (13.1 mg% and 10 mg%, resp.) (normal: 8.8–11 mg%; 2.5–4.8 mg%, resp.) along with increased levels of paratharmone (711.3 pg/mL; normal: 12–72 pg/mL). Ultrasound of neck revealed a well-defined hypoechoic lesion of 2.2 × 2 × 3.1 cm, located posteriorly and inferiorly to the right lobe of thyroid causing an indentation which was suggestive of a parathyroid adenoma. Skull radiographs revealed multiple well-defined osteolytic radiolucent lesions in the parietal and occipital areas (Figure 6).

Based on the clinical, radiographic, histological, and biochemical analyses, a final diagnosis of brown tumor associated with primary hyperparathyroidism was derived.

## 3. Discussion

Primary hyperparathyroidism is the 3rd most common endocrine disease [8], caused due to parathyroid adenomas, hyperplasias, or carcinomas [9]. Mostly it is a sporadic disease but may also occur in a familial pattern as autosomal dominant condition like hyperparathyroidism-jaw tumor syndrome (HPT-JT syndrome) and multiple endocrine neoplasia (MEN) syndrome [10].

HPT is commonly asymptomatic; however some patients may present with nonspecific symptoms like weight loss, GIT, and musculoskeletal disturbances [3] which was in concordance with our patient.

Classic skeletal lesions like bone resorption, bone cysts, brown tumors, and generalized osteopenia occur in less than 5% of all HPT cases [4]. The incidence of these skeletal lesions in HPT patients has fallen from 80% to 15% currently, which is attributed to better biochemical monitoring of calcium levels [5].

Brown tumor accounts for 10% of all skeletal lesions with a 0.1% incidence in jaws [5]. It is more common in females older than 50 years. Gender predilection may be attributed to hormonal imbalances which are common in females more than males [7]. In the present case also the patient was a 40-year-old female.

Brown tumor may involve any part of skeleton but is commonly seen in ribs, clavicle, and pelvis. In head and neck region, mandible is commonly involved compared to maxilla especially the posterior region [11]. The present case was also reported in the posterior mandible.

Symptoms caused by the lesion depend on their size and location. Clinically, brown tumor may present as small asymptomatic swelling in jaws or as a painful exophytic mass which was observed in the present case.

Radiographically brown tumors appear as a well-defined unilocular or multilocular radiolucent lesion with expansion of affected bone. Additional features include subperiosteal resorption of phalanges of index and middle fingers, generalized osteopenia, and focal areas of skull demineralization-salt and pepper appearance [12]. Similar changes were noted in skull radiograph of the present patient. In the jaws, radiolucent lesions are observed with altered trabecular pattern, root resorption, root displacement, and loss of cortication around inferior alveolar canal. A characteristic feature in the jaws is loss of lamina dura surrounding the roots of involved teeth which is also seen in this case [3].

Histopathologically brown tumor exhibits dense fibroblastic stroma, areas of cystic degeneration, osteoid, hemorrhage, macrophages with hemosiderin, and multinucleated osteoclastic giant cells [7]. Cystic appearance is due to intraosseous bleeding and tissue degeneration [5]. Similar features were reported in the present case also.

Histologically brown tumor mimics many other giant cell lesions of head and neck region. Clinical, radiographic, and histological features of giant cell mimickers are discussed in Table 1.

On biochemical investigations, the present case showed hypercalcemia and hyperphosphatemia, along with increased parathyroid hormone level which aided in the confirmatory diagnosis. These alterations may be due to elevated parathyroid hormone which activates the osteolytic pump causing loss of calcium from bone to extracellular fluid resulting in elevated serum calcium levels. Ultrasound, CT scan, or technetium scan techniques can also be used to detect the diseased parathyroid gland [10]. Ultrasound of our patient revealed a hypoechoic lesion lateral to thyroid gland suggesting a parathyroid adenoma.

Treatment of HPT is the first step in the management of brown tumor. After parathyroid excision, if the jaw lesions are smaller in size, they tend to regress spontaneously, either completely or partially. If the lesion is large and disfiguring or if the affected bone is weakened, surgical excision of the brown tumors is indicated. Some suggest systemic corticosteroids initially to decrease the size, followed by surgical excision of the residual lesion [11]. Recurrence is very rare once the hormonal levels revert back. Prognosis of the lesion mainly depends on the evaluation of biochemical parameters after extirpation of parathyroid tumor.

Even though the advancement of various diagnostic process and biochemical tests aids in early diagnosis of HPT, dentists should be aware of possible occurrence of brown tumor involving the jaws of undiagnosed patients as it may be presenting as the first manifestation. Hence it is essential that dentist should have the knowledge about oral manifestations associated with various systemic diseases leading to their early diagnosis.

## 4. Conclusion

Although the diagnosis of asymptomatic primary hyperparathyroidism is indicated by detection of elevated levels of calcium on routine biochemical analysis, still there is a possibility of patients presenting with advanced bony lesions. Therefore all giant cell lesions occurring in the jaws have to be further evaluated biochemically to rule out primary hyperparathyroidism.

## Figures and Tables

**Table 1:** Differential diagnosis of giant cell lesions.

S. number	Name of the lesion	Clinical features	Radiographic features	Histological features	Biochemical analysis		
					PTH	Ca	P
(1)	*Primary hyperparathyroidism (present case)*	*Older aged women are commonly affected by predilection for mandible*	*Unilocular or multilocular radiolucency*	*Numerous multinucleated giant cells, areas of hemosiderin, and osteoid are seen*	✓	✓	✓
(2)	Central giant cell granuloma	Common in younger individuals and occur in the anterior region of the jaw	Unilocular or multilocular radiolucency	Prominent but not numerous multinucleated giant cells, groups of collagen fibers, numerous foci of extravasated blood, and hemosiderin	—	—	—
(3)	Giant cell tumor or osteoclastoma	Common in third decade of life	Unilocular or multilocular radiolucency	Giant cells are scattered uniformly; areas of necrosis are seen	—	—	—
(4)	Aneurysmal bone cyst	Younger individuals	Multilocular with honeycomb or soap bubble appearance	Cavernous or sinusoidal blood filled spaces, multinucleated giant cells, hemosiderin pigment, and new osteoid formation are seen	—	—	—
(5)	Noonan-like multiple giant cell lesion syndrome	Autosomal dominant multiple congenital anomaly disorder, characterised by short stature, craniofacial dysmorphisms, and congenital heart defects (CHD)	Multilocular radiolucency	Numerous multinucleated giant cells, spindle shaped fibroblasts, and perivascular cuffing are seen	—	—	—
(6)	Cherubism	Painless, symmetric jaw lesions involving common maxilla	Multilocular radiolucencies with ground glass appearance	Numerous multinucleated giant cells, spindle shaped fibroblasts, and perivascular cuffing are seen	—	—	—
